# Reconfigurable Slippery Gradient Structure for Programmable Droplet Self-Propulsion

**DOI:** 10.1021/acs.langmuir.6c02822

**Published:** 2026-08-08

**Authors:** Ryo Sakai, Shunto Arai, Takashi Hiroi, Ryota Tamate, Timothée Mouterde, Mizuki Tenjimbayashi

**Affiliations:** † Research Center for Materials Nanoarchitectonics (MANA), 52747 National Institute for Materials Science (NIMS), 1-1 Namiki, Tsukuba, Ibaraki 305-0044, Japan; ‡ Department of Applied Chemistry, 70651 Chuo University, Tokyo 112-8551, Japan; § Research Center for Macromolecules & Biomaterials, NIMS, 1-1 Namiki, Tsukuba 305-0047, Japan; ∥ Department of Applied Chemistry, Graduate School of Engineering and Science, Shibaura Institute of Technology, Fukasaku 307, Minuma-ku, Saitama 337-8570, Japan; ⊥ Department of Mechanical Engineering, The University of Tokyo, 7-3-1 Hongo, Bunkyo-ku, Tokyo 113-8656, Japan

## Abstract

Transporting millimeter-sized droplets on a surface is fundamental for droplet fluidics, which requires minimization of droplet sticking and energy input. One promising strategy is tethering the self-propelling ability to droplets on nonsticking surfaces. Self-propulsion is achieved by forming a gradient structure on the substrate. Typically, the gradient is highly directional and nonreprogrammable, making the regulation of transport challenging. Here, we report that droplets are self-propelled from the edge to the center of the substrate. The substrate surface is covered with a stabilized lubricant that exhibits droplet slipperiness. Near the substrate edge, a lubricant thickness gradient forms, which self-propels the droplet. This property enabled the regulated self-propulsion of droplets by spatiotemporal edge-shape design. The edge shape determines the direction of the droplet’s self-propulsion. Moreover, the in situ edge appearance/disappearance, achieved by separating/connecting two neighboring coated substrates, enabled the regulation of self-propelling timing because the lubricant gradient structure is reconfigurable. We demonstrate droplets’ self-climbing, upside-down self-propulsion, in situ stop-and-go motion, two-droplet attractive motion, and reconfigurable two-dimensional droplet motion. We also demonstrate that the sliding droplet on the zigzag-shaped substrate moves along with the substrate’s shape due to edge self-propulsion. These droplet manipulations require no special devices, making them increasingly accessible.

## Graphical abstract

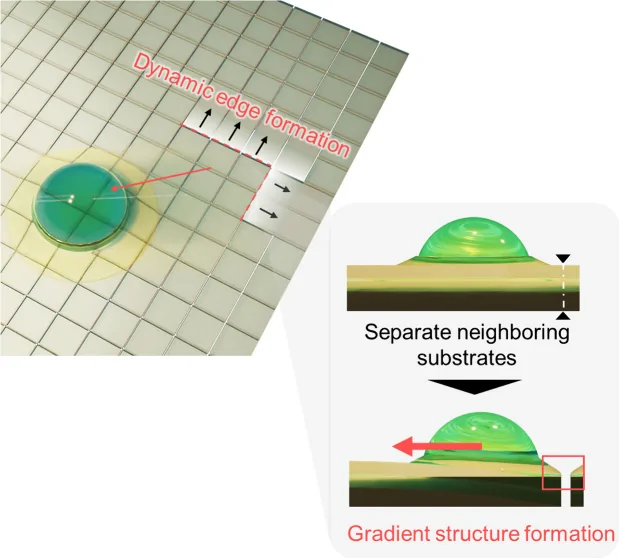

## Introduction

Droplet manipulation on a surface plays a crucial role in droplet fluidics applications, such as microreactors and harvesting.^1–4^ Droplets stick to the contacting media via contact lines, which decrease droplet mobility and hinder their transport. Various strategies have been adopted to improve droplet transport efficiency.^4^ One strategy is to apply the driving force to the droplet on a nonsticking surface, which requires an energy converter that converts noncontacting external stimuli into the driving force. The transducing function is equipped in droplets or nonsticking surfaces, which are triggered by a magnetic field,^5^ photoirradiation,^6,7^ ultrasonic,^8^ or heating.^9^ Despite the requirement for a large and/or expensive specialized device to trigger the transducer, this strategy enables in situ programmable droplet manipulation. Another strategy is to form a gradient structure and/or chemistry around the droplet,^10,11^ allowing it to self-propel along the preset gradient. For example, a droplet on a surface with a macroscopic anisotropic structure self-propels in a direction due to the Laplace pressure difference between its front and rear sides.^12^ In the self-propelling approach, neither a transducer nor a specific trigger device is required for transporting droplets; however, the applicable motion is monotonic, and flexible droplet manipulation is challenging. Considering the advantages of these strategies, one ideal strategy may be the design of a flexibly programmable and repeatedly self-propelled droplet on a versatile, nonsticking interface. To achieve this, the design of the in situ reprogrammable gradient structure may be promising.

Here, we selected the lubricant-impregnated surface (LIS)^13–15^ as the nonsticking platform for droplet manipulation. LIS exhibits nonsticking properties to the droplet that are immiscible with the lubricant. When the lubricant preferentially wets and remains immobilized on the substrate in the presence of a droplet, the droplet rests on the lubricant layer without direct contact with the underlying substrate, a phenomenon known as oleoplaning.^16^ In this state, the droplet is highly mobile, with no apparent contact-line pinning. Previous studies have explored various strategies for droplet manipulation on LIS.^1,3^ In particular, gradient interfaces have been created by shaping the lubricant-infused substrate into a gradient geometry,^17,18^ applying external gradient fields,^19,20^ patterning two different lubricants,^21^ making a gradient by varying the amount of infused lubricant,^22^ and forming lubricant menisci.^23–25^ These approaches have successfully enabled directional droplet self-propulsion.

Further efforts have focused on regulating the self-propulsion behavior, including furcated self-propulsion,^9,27^ study of the self-propelled droplet stop position,^28,29^ and coalescence of two droplets,^30^ tuning the velocity of the self-propulsion.^31^ Moreover, by controlling the gradient morphology of the lubricating base layer, the self-propelling speed and distance can exceed 30 mm/s and 30 mm, respectively, without external energy input.^31^

Here, we demonstrate droplet self-propulsion with “repeatedly” programmable two-dimensional trajectories and even propelling timing without relying on a special device. We noted the shape of the lubricant layer on the LIS-coated substrate. As long as the lubricant is not absorbed into the substrate, the lubricant layer forms a puddle shape. At the edge of the puddle, a thickness gradient forms due to the competition between the lubricant surface tension and gravity. We found the droplet self-propelled from edge to center in the thickness-gradient region, which led us to the idea of programmable self-propulsion by designing “edge shapes and in-situ appearance”. Owing to the fluidic nature of the lubricant, the gradient lubricant thickness is reconfigurable depending on the edge structures. We show edge shape variation and in situ edge formation by combining several LIS-coated substrates, enabling the spatiotemporal programming of droplets’ self-propulsion. Edge formation is programmable flexibly/repeatedly simply by positioning the LIS-coated substrate, and neither a special device nor a functional interface is required.

## Results and Discussion

### Edge-to-Center Self-Propulsion

We first examine the droplets’ self-propulsion. The model LIS was prepared by immobilizing silicone oil on the phenyl silane-modified non-textured glass substrate.^32,33^ To eliminate the evaporation effect, nonvolatile ionic liquid 1-(2-hydroxyethyl)-3-methylimidazolium tetrafluoroborate with surface tension of γ_drop_ = 58 mN/m and density of 1.34 g/cm^3^ is used as a probe droplet. Our previous works confirmed the stability and nonsticking property of the LIS.^32,33^ The experimental setup is shown in Figure 1(a). We cast 1 μL of ionic liquid droplet on the edge of the LIS. We defined *D* as the distance between the substrate-edge side end of the droplet and the substrate edge. We also varied the lubricant amount to change its thickness *ε.* ^33^ Note that the lubricant thickness varied with the substrate position. Thus, we also defined the lubricant thickness in the plateau region *ε*_plateau_, that is determined from side-view microscopic images. The cast droplet exhibited self-propelling behavior from the edge to the center of the LIS-coated substrate, as shown in Figures 1(b), 1(c), and Movie S1. We noticed that the droplets’ self-propulsion velocity d*D*/d*t* is larger on the LIS with *ε*_plateau_ ≈ 380 μm than on the LIS with *ε*_plateau_ ≈ 110 μm. We plot in Figure 1(d) the time *t* dependence of *D* for different *ε*_plateau_, from which we find that the self-propelling distance increases with the lubricant thickness. Here, we obtained the power-law dependence *D* ∼ *t* ^α^ with α ≈ 0.24 ± 0.02 from the slope of the double-logarithmic plot in Figure 1(e), as discussed in section “Self-propelling mechanism”. We further plotted the time evolution of the velocity d*D*/d*t* in Figure 1(f), confirming that the droplet velocity increases with the lubricant thickness at the initial stage and then exponentially decreases with *t*. The decrease in velocity indicates that frictional forces counteract droplet propulsion.

**Figure 1:**
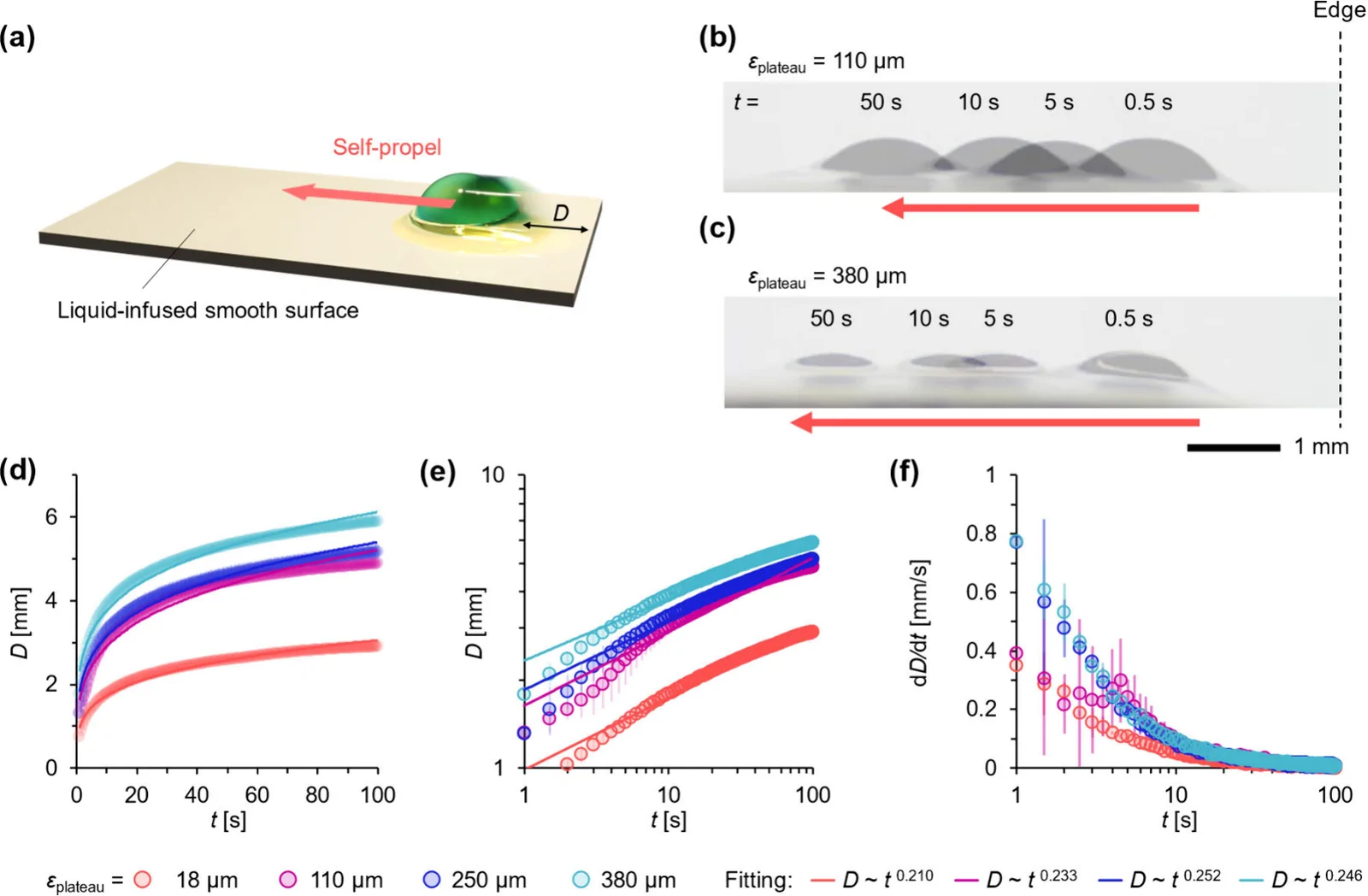
(a) Droplet self-propulsion behavior. We define the distance between the substrate-edge side end of the droplet and the substrate edge as *D*. (b and c) Time-lapse snapshots of self-propelled droplets for plateau-region lubricant thicknesses *ε*_plateau_ of 110 and 380 μm, respectively. (d and e) Time evolution of droplet position *D* for different lubricant thicknesses ranging from 18 to 380 μm on a linear scale and log–log axis, respectively. (f) Time evolution of self-propelling velocity d*D*/d*t* plotted on a semilog axis.

### Self-Propulsion Mechanism

We study the mechanics of the droplet self-propulsion. We first confirmed that the driving force is applied to the droplet in the region of the gradient thickness area. We quantified the force applied to the droplet as it approached the edge using the cantilever method.^16,33^ The experimental setup is shown in Figure 2 (a). The probe droplet is cast on the center of the LIS-coated substrate and attached to the capillary tube. Then, the substrate is translated to make the droplet approach the edge (decreasing *D*) at the speed of *U* = 1 mm s^–1^. When a force *F* is applied to the droplet, the capillary tube deflects by a distance δ, and we can estimate the applied force using Hooke’s law *F* ≈ *kδ,* where *k* = 2.8 μN/mm is the spring constant of the capillary tube. Figure 2(b) shows the capillary deflection as *D* decreases. We find that the force required to propel the droplet increases as *D* decreases.

**Figure 2:**
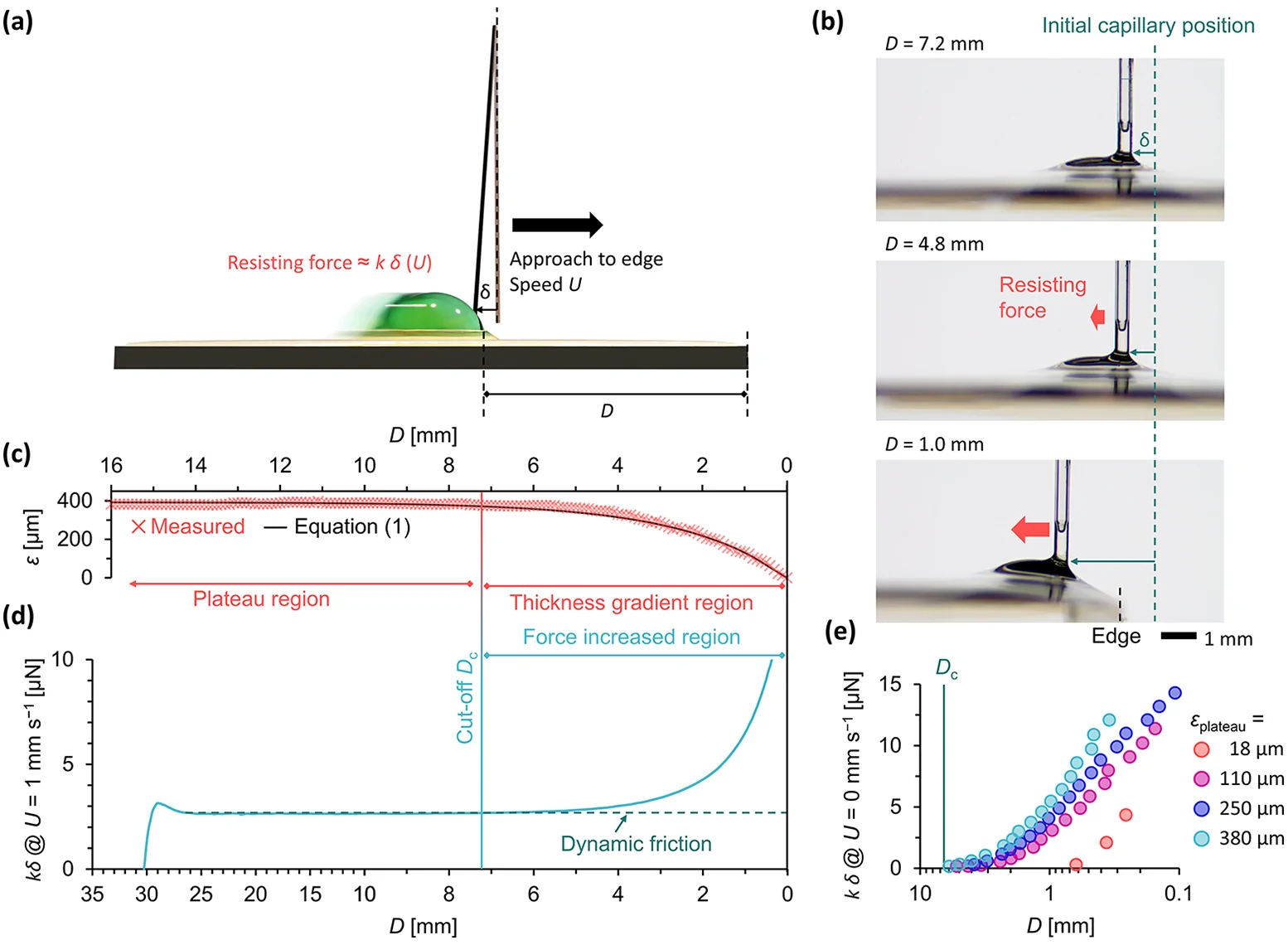
(a) Scheme of experimental configurations for measuring the self-propulsion region. The droplet on the liquid-infused surface is attached by a capillary tube and approaches the edge. The deflection of the capillary tube indicates the force applied to the droplet. (b) Capillary tube deflection for different droplet–edge distances. (c) Lubricant thickness profile for a plateau region with a thickness of 380 μm. (d) Resisting force in the droplet approaching the edge at a speed of *U* = 1 mm s^–1^. The plateau force region corresponds to dynamic friction due to viscous dissipation. (e) Static components of driving force applied to the droplet, which is obtained by the capillary deflection without motion (*U* = 0 mm s^–1^).

We evaluated and compared how the thickness *ε* and the force *F* = *kδ* depend on *D* in Figures 2(c) and 2(d) on the LIS with *ε*_plateau_ ≈ 380 μm. As shown in Figure 2(c), *ε* obtained from side-view microscopic images increases with *D* and approaches the plateau value (that is, *ε* → *ε*_plateau_). The apparent thickness gradient region is observed at *D* < 7–8 mm. The competition between the lubricant surface tension and gravity forms this gradient. In the competing area and assuming small deformations (|d*ε*/d*D*| ≪ 1), the Laplace pressure jump at the lubricant surface – γ_lub_d^2^ *ε*/d*D* ^2^ is equal to the hydrostatic pressure *ρg*(*ε*_plateau_ – *ε*), where γ_lub_, ρ, and *g* are, respectively, the lubricant surface tension, density, and gravitational acceleration constant. That is equivalently d^2^ *ε*/d*D* ^2^ = κ^2^(*ε* – *ε*_plateau_), where κ^–1^ = (γ_lub_/*ρg*)^1/2^ ≈ 1.56 mm is the capillary length of the lubricant. Here, the boundary conditions are *ε* → *ε*_plateau_ when *D* → + ∞, and *ε* → 0 when *D* → 0. By solving this, we obtain the lubricant thickness distribution to be

$$\varepsilon =\varepsilon_{\mathrm{plateau}}\left[1-\exp\left(-D\kappa \right)\right]$$ 1

where κ^–1^ is the capillary length of the lubricant. The model fits well with the measured value in Figure 2(c). Here, *ε* exceeds 99% of *ε*_plateau_ (that is, the region that can be regarded as sufficiently flat) at around *D* ≈ 7.2 mm, which is comparable to the distance over which motion was experimentally observed. We define the cutoff length *D*_c_ ≈ 7.2 mm. Note that Equation 1 indicates that *D*_c_ does not depend on *ε*_plateau,_ contrary to the thickness gradient d*ε*/d*D* = *κε*_plateau_ exp­(−*Dκ*), which is proportional to *ε*_plateau_. Considering that the thickness gradient may propel the droplet, we expect the self-propelling driving force to act on the droplet for *D* < *D*_c_. Figure 2(d) and Figure S1 show the variations of the force *kδ* with decreasing *D*. Upon starting sliding at *D* ≈ 30 mm, the droplet first experiences static friction. Then, the force transitions to a plateau corresponding to dynamic friction due to viscous dissipation.^16,33,34^ Finally, in the thickness gradient region (*D* < *D*_c_), the force is observed to exponentially increase with the decrease of *D*. In other words, the resisting force acting on the droplet increased at *D* < *D*_c_ as it approaches the edge, as expected. Moreover, the resisting force does not depend on the velocity. We observed the capillary deflection of the resting droplet (that is, *U* = 0 mm s^–1^) with different *D* and *ε*_plateau_ as displayed in Figure 2(e). We observed that the static force applied to the droplet increases with the decrease of *D*. In comparing the force curve at *ε*_plateau_ ≈ 380 μm with Figure 2(d), we find the static force at *U* = 0 mm s^–1^ is approximately the same as the increased force at *U* = 1 mm s^–1^ and *D* < *D*_c_, which means the friction increase in Figure 2(d) is static force component. Moreover, Figure 2(e) indicates that the static force components increase with *ε*_plateau_ for all *D*.

Then, we return to the case in which the droplet is free to move on the substrate to investigate the origin of the driving force for the self-propelled droplet. Figures 3(a) and 3(b) illustrate the interfacial states of the droplets with different *D* obtained from their side-view photos. When the droplet is placed in a plateau thickness region far from the edge (that is *D* > *D*_c_), the droplet shape is symmetric, and the lubricant wedge angles Θ_c_ and Θ_e_ defined, respectively, as the angles facing the center or the edge of the substrate, are similar (Θ_c_ **≈** Θ_e_) as illustrated in Figure 3(a). In contrast, when the droplet is near the edge, the wedge shape is asymmetric, and we find Θ_c_ **≪** Θ_e_ owing to the lubricant thickness gradient. This wedge shape difference is observed in the self-propelled droplet with different *D* (Figures 1(c), 3(c), and 3(d)). Here, we define the apparent droplet radius *R* and the wedge height of the lubricant *H*(*ε*). Note that *H*(*ε*) is the sum of *ε* and the increased height of the lubricant by cloaking the droplet. We assume that Θ_c_ is constant because it is far enough from the edge. While Θ_e_ is apparently larger than Θ_c_ due to the edge-induced thickness gradient. We assume that Θ_e_ is given by the static wedge angle Θ_c_ tilted by the local interface angle tan^–1^[*H*(*ε*)/*D*], and we obtain

$$\Theta_{e}=\Theta_{c}+\tan^{-1}\left[H\left(\varepsilon \right)/D\right]$$ 2

**Figure 3:**
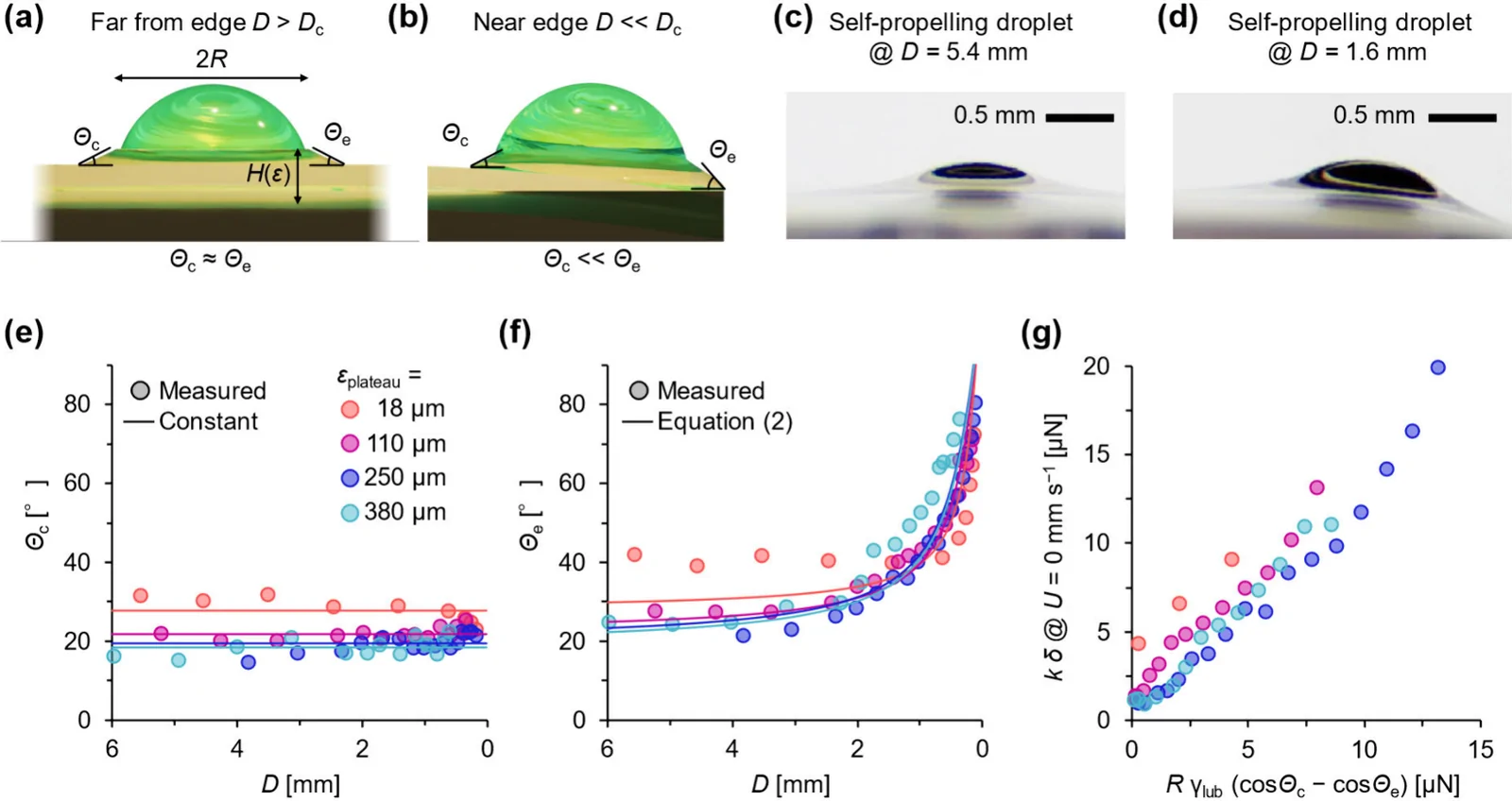
(a and b) Side-view schemes of the droplet on the LIS-coated substrate with *D* > *D*_c_ or *D* ≪ *D*_c_. (c and d) Selected snapshots of the self-propelled droplets at different *D* values. (e and f) Lubricant wedge angles as a function of the droplet–edge distance. (e) Front and rear side wedge angle relative to the self-propulsion direction and (f) rear side one (close to the edge). (g) Correlation of the driving force obtained by capillary deflection with the estimated capillary force induced by wedge angle hysteresis.

Note that *H*(*ε*) tends to increase with the *ε*_plateau_ (See Figure S2). Figures 3(e) and 3(f) quantified the angle evolution with *D*, which showed good agreement with our estimation. Here, the driving force to propel the droplet (≈ *kδ* at *U* = 0 mm s^–1^) may be related to the anisotropic wedge shape. Because the droplet is cloaked by lubricant (Figure S3), the capillary force owing to the anisotropic wedge shape yields *Rγ*_lub_(cosΘ_c_ – cosΘ_e_). Figure 3(g) plots *kδ* at *U* = 0 mm s^–1^ as a function of *Rγ*_lub_(cosΘ_c_ – cosΘ_e_), which exhibits a linear proportion. Thus, we have *kδ* ∼ *Rγ*_lub_(cosΘ_c_ – cosΘ_e_) at *U* = 0 mm s^–1^.

We then discuss the power-law dependence *D* ∼ *t* ^0.24^ that we observed in Figure 1(e). We considered that the self-propelling force ∼ *Rγ*_lub_(cosΘ_c_ – cosΘ_e_) is resisted by viscous dissipation in the lubricant, which scales as γ_lub‑drop_ *R*Ca^2/3^, with Ca the capillary number Ca *= η*/*γ*_lub‑drop_d*D/*d*t*, *γ*_lub‑drop_ is lubricant–droplet interfacial tension, and η the lubricant viscosity.^16,33,35^ We first observe that Θ_c_ **≪** Θ_e_ **<** 1 at small *D,* and considering the lower-order terms in the Taylor expansion of cosine, we can rewrite capillary force γ_lub_ *R*(cosΘ_c_ – cosΘ_e_) as γ_lub_ *RΘ*_e_^2^/2. Taking the first-order Taylor expansion of eq 2, we get Θ_e_ **≈** tanΘ_e_ **≈** *H*(*ε*)/*D*, so that the capillary force scales as γ_lub_ *RH*(*ε*)^2^ */D* ^2^. Balancing this force with the viscous dissipation γ_lub‑drop_ *R*Ca^2/3^, we get *D* ^3^d*D*/d*t* ∼ [γ_lub_^3/2^/(γ_lub‑drop_^1/2^ η)]*H*(*ε*)^3^ which yields *D* ∼ [γ_lub_^3/8^/(γ_lub‑drop_^1/8^ η^1/4^)] *H*(*ε*)^3/4^ *t* ^1/4^. Therefore, the time dependence of the position scales as *D* ∼ *t* ^1/4^; this value is in good agreement with the scaling observed in Figure 1(e).

### Edge-Induced Droplet Manipulation

The self-propelled droplet near the edge of the LIS-coated substrate enabled flexible droplet manipulation. Figure 4 demonstrates one-dimensional droplet manipulation. Figure 4(a) and Movie S2 show the droplet attached to the LIS-coated substrate with upside-down self-propelled. The droplet is not detached by gravity because the cloaked lubricant layer prevents it from being pulled down. Moreover, the droplet climbs the 2° tilted substrate (Figure 4(b) and Movie S3), indicating that the self-propelling driving force remains active as long as the lubricant thickness gradient is maintained.

**Figure 4:**
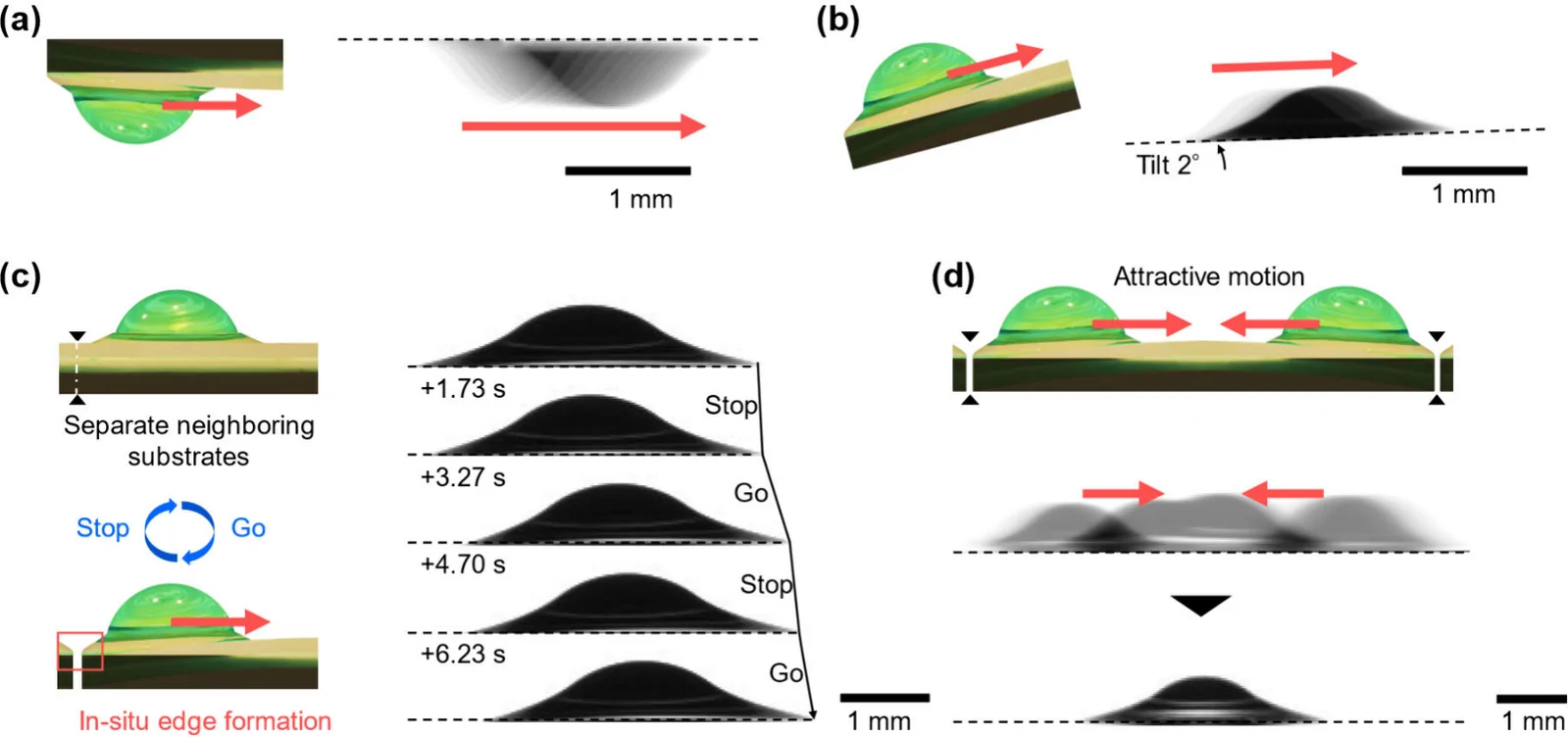
One-dimensional droplet manipulation. (a) Self-propelled droplet on the upside-down substrate. (b) Self-climbing on tilted substrates. (c) Cyclic droplet propelling/stopping motion by in situ substrate edge formation. Two coated substrates are connected, and the droplet is placed near the border of the two substrates. When the neighboring substrates are separated, the droplet starts motion and stops when the substrates contact again. (d) Separation of neighboring substrates initiates the mutual attraction between two droplets. After making contact, the two droplets coalesced into one.

We then show advanced droplet manipulation using several substrates. The design principles are as follows. When two LIS-coated substrates are joined together to form a pair, the lubricant thickness is uniform at their interface. Once these substrates are separated, lubricant thickness gradients form at the newly created substrate edges. The substrates’ separation/attachment action reversibly changes the lubricant thickness profiles and triggers the droplets’ self-propulsion. Using this principle, Figure 4(c) and Movie S4 demonstrate the droplet’s repeated stop-and-go motion. We continuously attached/separated neighboring LIS-coated substrates.

Along with these actions, the droplet exhibited remarkably sensitive self-propulsion. The sensitive droplets’ response arises from the quick recovery of the lubricant thickness profile. Moreover, we show the coalescing behavior of the two droplets in Figure 4(d) and Movie S5. In this demonstration, the edge formation triggers the attractive motion of two droplets. Once these droplets are close, the lubricant wedge brings them into contact with each other, akin to the Cheerios effect,^23,24,36^ and the droplets finally coalesce as the intervening lubricant layer is squeezed out.

Finally, we demonstrate two-dimensional droplet motion in Figure 5. The setup for two-dimensional manipulation is shown in Figure 5(a). We prepared several LIS-coated, rectangle-processed substrates with their long sides attached. Note that static driving forces are applied to the droplet from the edges of these substrates, which decrease with *D*. By prepositioning how these substrates attach to one another, the edge distribution can be designed in two dimensions. Figure 5(b) and 5(c), and Movie S6, exhibit diagonal movement at different angles. The inserted arrows in these chronophotographies indicate the potential driving forces applied to the droplet. The only difference between these figures is the force to resist the diagonal motions (highlighted by blue-colored arrows). Owing to the different *D*, the forces resisting the diagonal motions for the case in Figure 5(c) are weaker than those in Figure 5(b); thus, the diagonal angle is larger. This experiment shows the regulated self-propulsion of the droplet with two-dimensional freedom.

**Figure 5:**
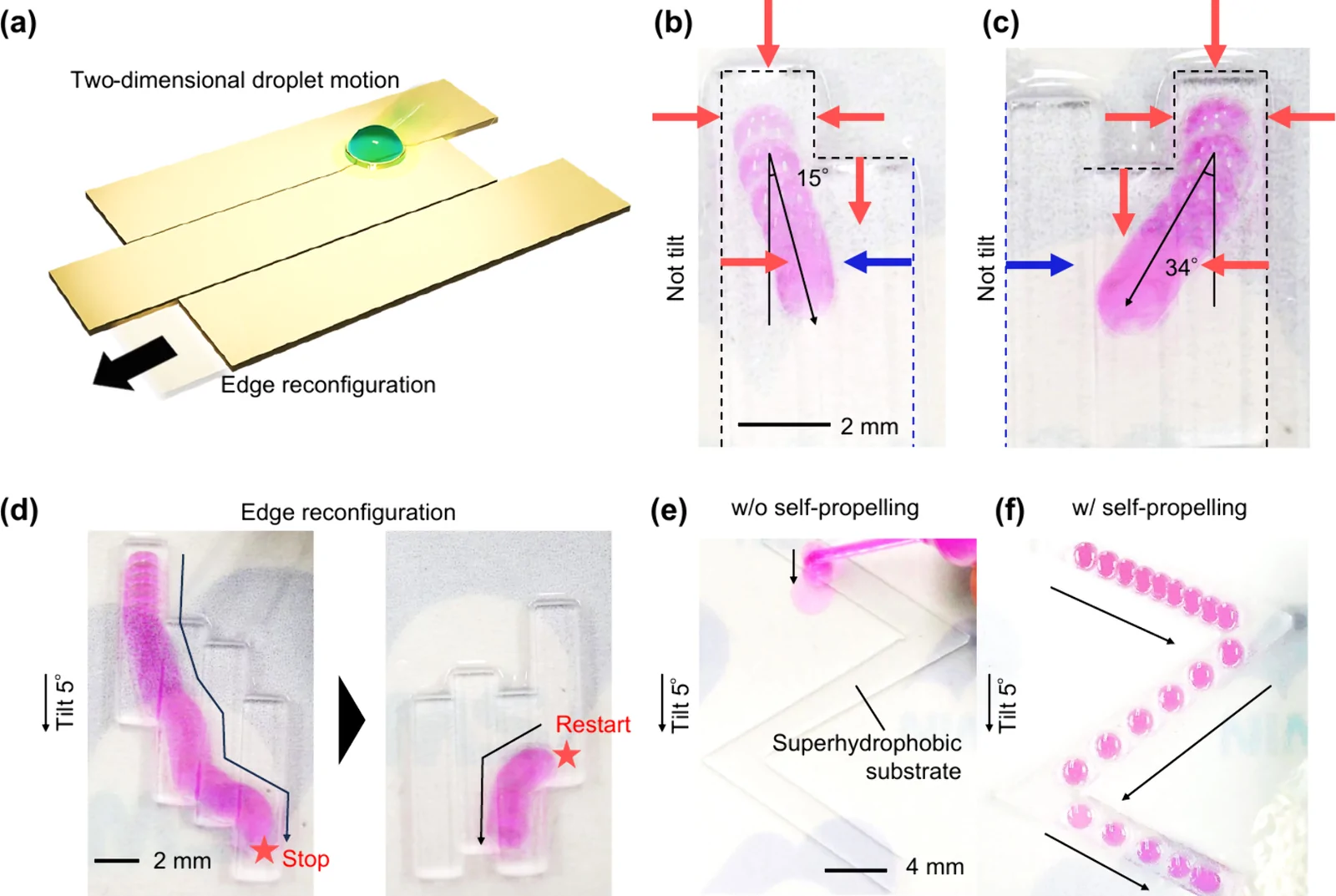
Two-dimensional droplet manipulation. (a) Scheme of setups. The processing and/or connection of the coated substrates enables flexible edge positioning. (b–f) Experimental photographs of droplet manipulation. (b and c) Diagonal self-propulsion with different angles of 15° and 34°, respectively, diagonal due to the difference in the capillary force applied from the blue dotted edge. (d) Edge reconfiguration-based in situ programmed droplet sliding. Four elongated coated substrates are connected in parallel. The plates slide to regulate the edge shape. In the initial arrangement of the substrates (left), the droplet slides from the top left to the bottom right and stops. Then, the substrates are arranged as shown in the right photos. The droplet restarted, sliding to the opposite side. (e and f) Droplet sliding motion on zigzag processed substrates with different surface wettabilities: superhydrophobic and liquid-infused smooth surfaces, respectively. On the superhydrophobic surface, the droplet slides straight down in the direction of the tilt. On the liquid-infused smooth surface, the droplet slides down along the substrate surface owing to self-propulsion from the edge.

The edge shape is dynamically reconfigurable; thus, edge reprogramming is possible. Figure 5(d) and Movie S7 show the reprogrammable two-dimensional droplet motion supported by gravity. In the first edge distribution, as shown in the left photo in Figure 5(d), the droplet is transported from the top left to the bottom right and stops due to edge self-propelling-assisted sliding. Then, the droplet restarted two-dimensional motion, as shown in the right panel of Figure 5(d), when we varied the substrates’ attachment modes via edge reconfiguration.

Lastly, we demonstrate that complex-shaped substrates make manipulation increasingly flexible. Figure 5(e), 5(f), and Movie S8 show the droplet sliding motion on zigzag-shaped substrates. One of the substrates is LIS-coated, and, for comparison, the other substrate is superhydrophobic-coated, as reported in our previous work.^37^ When the droplet is cast onto the superhydrophobic substrate, it rolls off in a tilted direction (Figure 5(e)). Conversely, the droplet on the LIS-coated substrate slides off along the substrate shape owing to the assistance of the edge self-propulsion (Figure 5(f)).

## Conclusion

This work reports edge-induced self-propulsion of droplets on a LIS-coated substrate, enabling flexible droplet manipulation by controlling the self-propulsion direction. The self-propelling force originates from capillary force due to anisotropy in the lubricant wedge around the droplet, increasing with lubricant thickness and decreasing droplet–edge distance. The lubricant gradient is not limited to our LIS but is also extensible to various LIS systems. Thus, the droplet is not limited to ionic liquids for this examination but can be water, oil, or other solvents by modulating LIS ingredients. The feature of this system is pre- and postprogrammable free two-dimensional motion. We examined the droplet manipulation ability as a function of the substrates’ edge design. The droplets’ self-propelling length is around 7.2 mm, and their speed reaches up to 0.8 mm/s, depending on the geometry of the gradient structure. The speed can be tuned by adjusting the lubricant amount; however, performance decreases with reduced lubricant thickness, which can be attributed to lubricant depletion.^38^ Thus, the method of supplying or preventing the loss of the lubricant layer^39–41^ may be critical to maximizing the performance of our strategy. We expect the systematic design of the (complex) substrate shape, the combination of upside-down substrates, and the synergetic use of other forces, such as droplet self-attraction and gravity, will make this technology increasingly flexible and versatile. High-quality substrate processing and precise substrate positioning can improve droplet manipulation accuracy. The accuracy of the manipulation may be improved by segmentation of the LIS-coated substrate. Since the suggested manipulation strategy is universal and does not require special devices, setups, or materials, it contributes to sustainable advanced microfluidics. Furthermore, the suggested mechanism can be combined with other self-propelling strategies.

## Supplementary Material



















## Notes

Self-propelled droplet from the edge to the center of the substrate (Movie S1) (MP4)
Self-propelled droplet attached to the substrate upside-down (Movie S2) (MP4)
Droplet self-climbing the 2° tilted substrate (Movie S3) (MP4)
Repeated stop-and-go motion of the droplet (Movie S4) (MP4)
Attractive motion of two droplets (Movie S5) (MP4)
Diagonal self-propulsion (Movie S6) (MP4)
Reprogrammable two-dimensional droplet motion (Movie S7) (MP4)
Droplet sliding motion on zigzag-shaped substrates (Movie S8) (MP4)
Method section, effect of lubricant thickness on the self-propulsion forces, effect on the wedge height, and lubricant wrapping of a droplet on the LIS (PDF)

## #

School of Advanced Science and Engineering, Waseda University, 3-4-1 Okubo, Shinjuku-ku, Tokyo 169-8555, Japan
