# Supplementary material for: Reconfigurable Slippery Gradient Structure for Programmable Droplet Self-Propulsion
Source: Langmuir. 2026 Aug 8;42(33):24168–75. doi: 10.1021/acs.langmuir.6c02822 (PMC13523735; doi:10.1021/acs.langmuir.6c02822)
Supplement: Supplementary file 1 [file la6c02822_si_009.pdf]

Reconfigurable slippery gradient structure for Programmable Droplet Self-propulsion

Ryo Sakai[1,2], Shunto Arai[3], Takashi Hiroi[4#], Ryota Tamate[3], Timothée Mouterde[5], and Mizuki Tenjimbayashi[1,2]**

[1]Research Center for Materials Nanoarchitectonics (MANA), National Institute for Materials Science (NIMS), 1-1 Namiki, Tsukuba, Ibaraki, 305-0044 Japan

[2]Department of Applied Chemistry, Chuo University, Tokyo 112-8551, Japan

[3]Research Center for Macromolecules & Biomaterials, NIMS, 1-1 Namiki, Tsukuba, 305-0047, Japan

[4]Department of Applied Chemistry, Graduate School of Engineering and Science, Shibaura Institute of Technology, Fukasaku 307, Minuma-ku, Saitama 337-8570, Japan

[5]Department of Mechanical Engineering, The University of Tokyo, 7-3-1 Hongo, Bunkyo-ku, Tokyo 113-8656, Japan

*Email: mouterde@g.ecc.u-tokyo.ac.jp

*Email: TENJIMBAYASHI.Mizuki@nims.go.jp

Caption of Movies

Movie S1 Self-propelling droplet from the edge to the center of the substrate.

Movie S2 Self-propelling droplet attached to the substrate upside-down.

Movie S3 Droplet self-climbing the 2° tilted substrate.

Movie S4 Repeated stop-and-go motion of the droplet

Movie S5 Attractive motion of two droplets

Movie S6 Diagonal self-propelling.

Movie S7 Reprogrammable two-dimensional droplet motion

Movie S8 Droplet sliding motion on zig-zag-shaped substrates

Method

All chemicals are used as received, and all experiments are conducted at room temperature (20−22°C).

LIS preparation: We first prepared lubricant immobilizing coating by infusing the plasma cleaned glass substrate (Micro slides, MUTO PURE CHEMICALS CO., LTD., Japan) with the coating mixture of 1 mL Phenyltriethoxisilane (Tokyo Chemical Industry Co., Ltd., Japan), 20 mL hexane (NACALAI TESQUE, INC.), and 1μL hydrochloric acid (FUJIFILM Wako Pure Chemical Corporation, Japan) for 24 h. After the immersion, the substrate was washed with acetone (NACALAI TESQUE, INC.), hexane, and ultrapure water (obtained using Direct-Q UV3 system, Merck KGaA, Germany). Then, silicone oil (X-22-170BX, Shinetsu Co., Ltd., Japan) is cast on the coated glass substrate to form LIS.

Droplet resisting force: We cast a droplet of ionic liquid 1-(2-Hydroxyethyl)-3-methylimidazolium tetrafluoroborate (IoLiTec Ionic Liquids Technologies GmbH, Germany) on the LIS. The droplet is attached to a cantilever (glass capillary with inner/outer radius of 0.18/0.30 mm, length of 300 mm, and the spring constant of 2.8 mN/m determined in our previous study[23]). Then, the substrate bottom is moved in parallel using a single-axis stage (SIGMA KOKI CO., LTD., Japan). The cantilever deflection and the droplet shape are observed using a USB camera (Swift Optical Instruments, USA).

Droplet self-propulsion: Droplet self-propulsion is observed using a USB camera. For demonstration, a droplet is dyed with Oil Red O (FUJIFILM Wako Pure Chemical Corporation, Japan) and observed using a digital camera (Nikon D5600, Nikon Co., Ltd.).

Superhydrophobic coating: Superhydrophobic coating is formed by hand-spraying 4wt.% dispersion of hydrophobic fumed silica nanoparticle (Aerosil RX200, Evonik industries, Germany) in Isopropanol (NACALAI TESQUE, INC.).

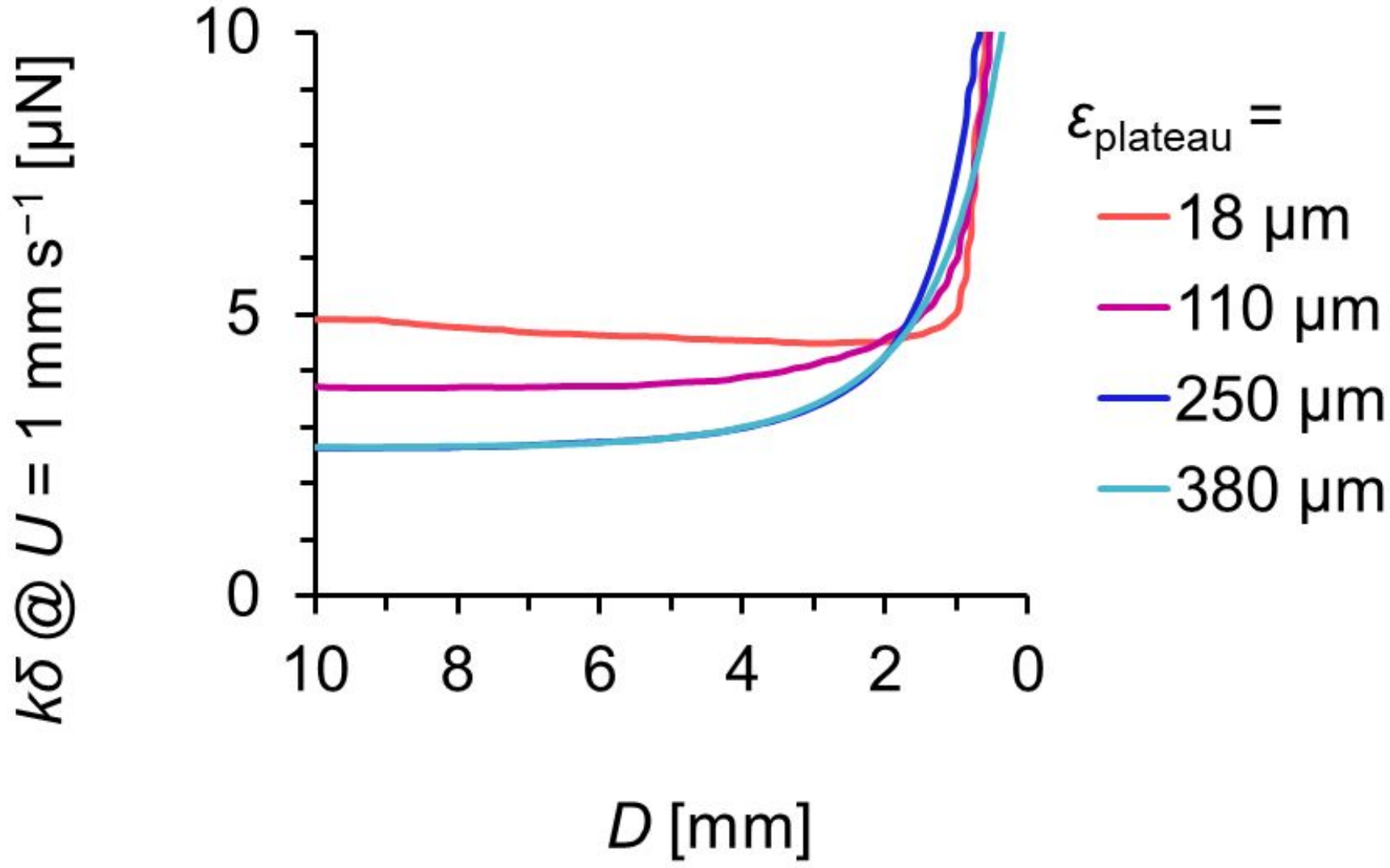

Figure S1. Resisting force in approaching droplet to the edge at the speed of $U = 1$ mm s^{-1} with different lubricant thickness.

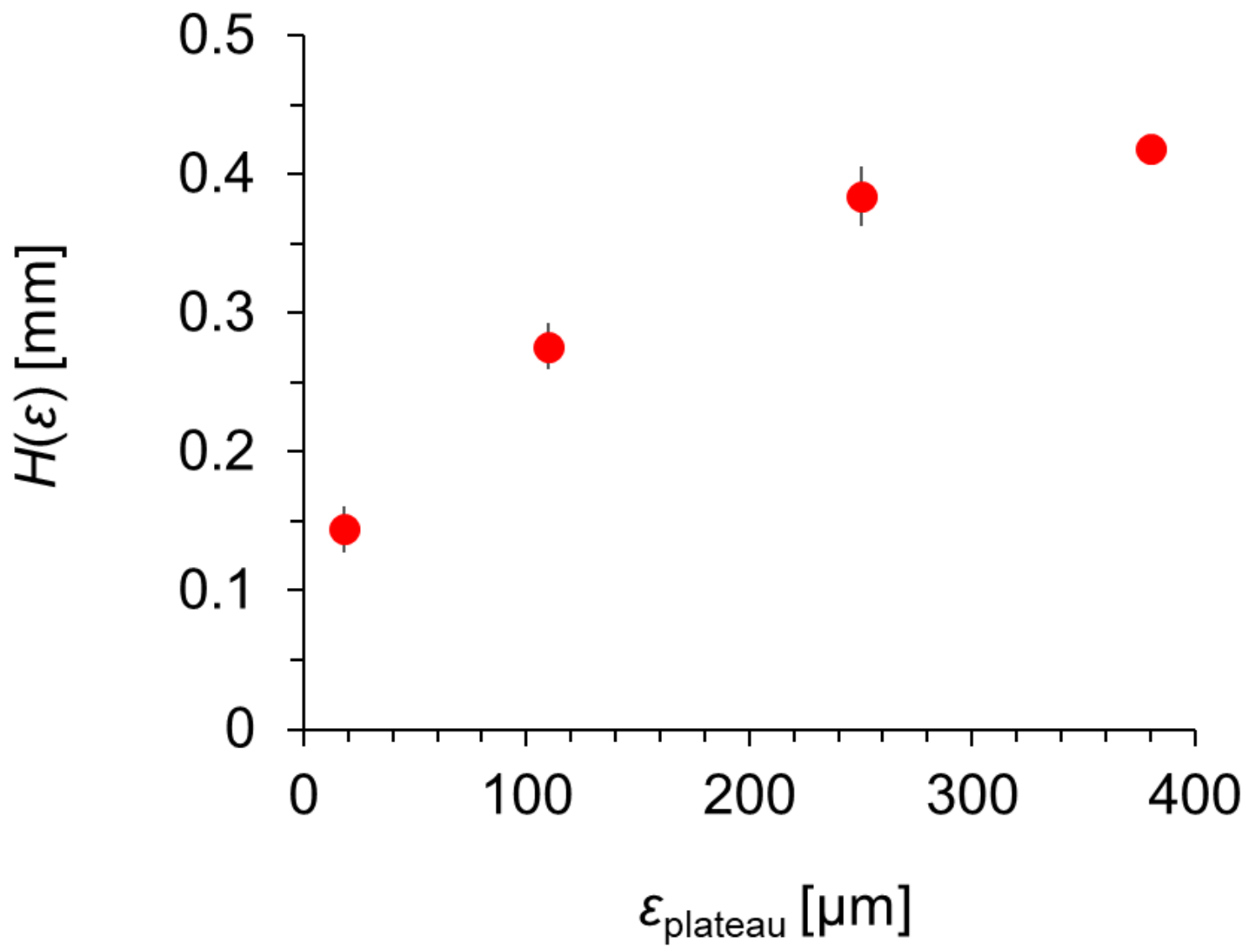

Figure S2. Wedge height of the lubricant as a function of plateau thickness.

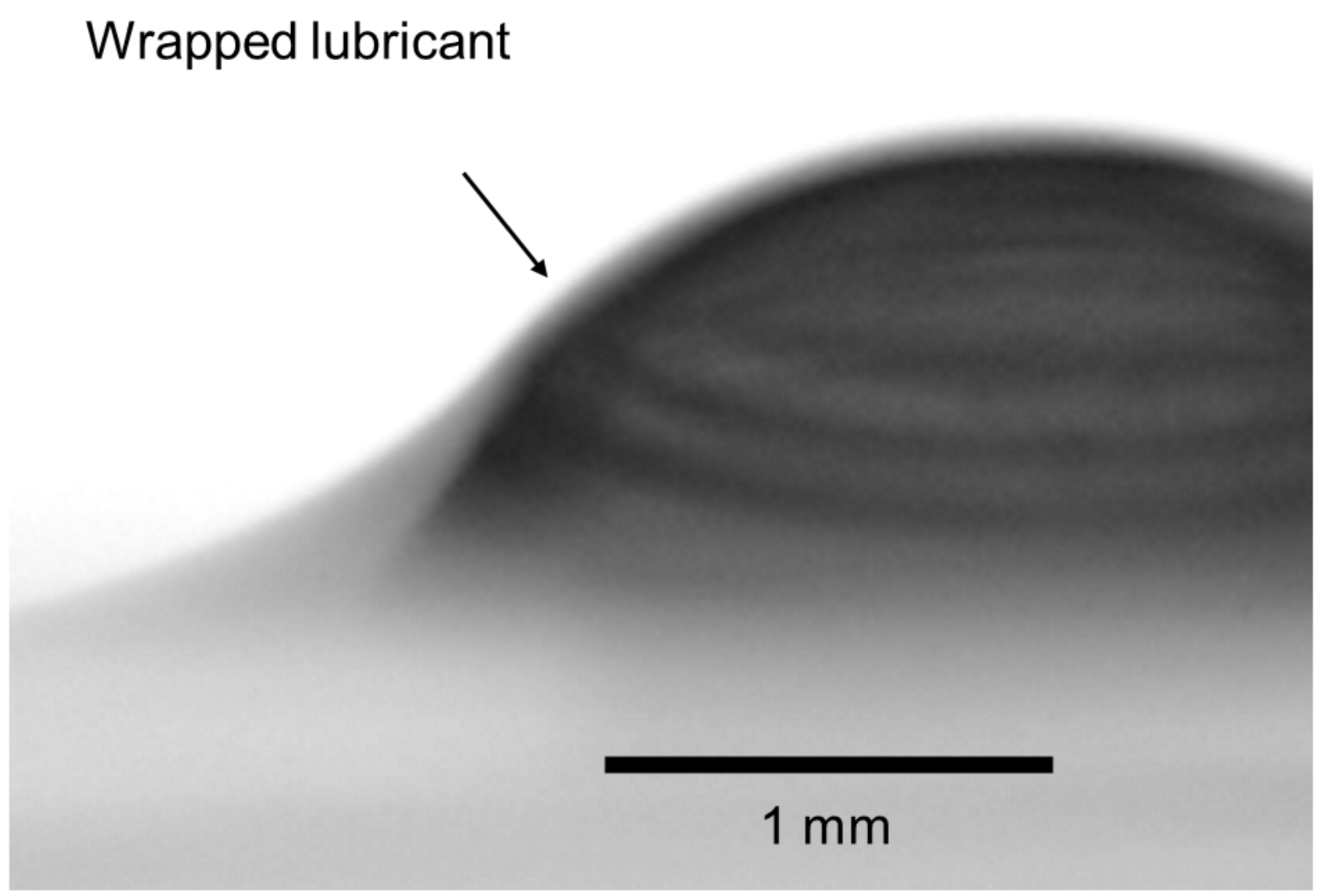

Figure S3. Lubricant wrapping of a droplet on LIS.
